# Bupropion Induced Hyponatremia in an Elderly Patient: A Case Report and Review of the Literature

**DOI:** 10.1155/2016/5103471

**Published:** 2016-06-28

**Authors:** Sahil Munjal, Yvette Smolin

**Affiliations:** New York Medical College, Westchester Medical Center, 100 Woods Road, Valhalla, NY 10595, USA

## Abstract

We present the case of a 72-year-old female with a major depressive episode who developed hyponatremia associated with bupropion. In reviewing the literature, there are only a few case reports which pertain to this topic. The clinical symptoms of hyponatremia can be misinterpreted as a worsening of the primary psychiatric illness and can lead to potentially serious consequences if not fully evaluated. We recommend that clinicians should be well aware of this side effect and that sodium levels should be checked within the first 2 weeks after initiating treatment in patients, especially those with additional risk factors for hyponatremia, such as older age, female sex, diuretic use, low BMI, and unexplained mental status changes at any time during treatment with antidepressants. The risk for hyponatremia associated with mirtazapine appears to be low and its use can be helpful in patients who have developed hyponatremia induced by other antidepressants and who experienced symptoms of weight loss and insomnia.

## 1. Introduction

Bupropion is an antidepressant which acts by inhibiting the reuptake of dopamine and noradrenaline. Although hyponatremia has been reported to be associated with use of various antidepressants, especially selective serotonin reuptake inhibitors (SSRIs) and serotonin norepinephrine reuptake inhibitors (SNRIs), it has rarely been reported with bupropion. Some authors hypothesize that hyponatremia is due to an antidepressant's potency to inhibit the reuptake of serotonin, thought to be due to a serotonin-induced increase in ADH, and mediated by the hypothalamic serotonin receptors. Alternatively, the limited evidence of bupropion as a causative agent of hyponatremia suggests that the mechanisms by which antidepressants can provoke hyponatremia may not only be related to their potential to inhibit serotonergic reuptake.

## 2. History of Present Illness

Ms. A was a 72-year-old single female, referred by her primary care provider (PCP) for evaluation of worsening depression. The patient was diagnosed with depression in her early twenties and was reportedly stable since, being productive and working as a clerk for fifty years. She never married and lived alone. She retired last year and quickly began to get more depressed and socially withdrawn, not eating with an unintentional weight loss of over 30 pounds in the past few months and overall not caring for herself. She reported anxiety related to her finances despite being financially secure. She had passive suicidal ideation of putting a cover over her head and “just going to sleep.” She admits having feelings of hopelessness, poor sleep, poor appetite, and despair related to not getting better. She now weighs about 80l bs, down from her baseline of 110 pounds. She denied any history of substance use or any manic symptoms. In the past four months, her PCP prescribed escitalopram which was titrated from 10 mg to 20 mg daily and alprazolam 0.25 mg tid, resulting in minimal clinical response. Pt. was not on any other medications at the time of admission.

## 3. Course of Hospitalization

**Figure 1:**
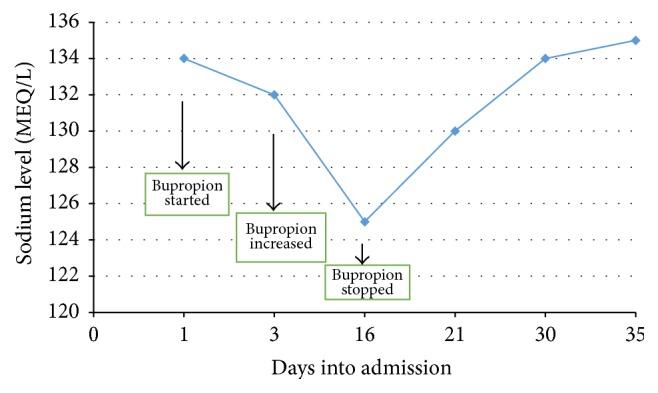
Serum sodium levels through the hospital stay.

Patient was started on bupropion 37.5 mg PO BID with clonazepam 0.25 mg BID to target the symptoms of depression and anxiety. Pt. was tolerating the medication well and the dose was titrated up to 75 mg BID in a few days. Patient's sodium level at admission was 132 MEQ/L (135–150). Also, her other basic laboratory tests were within normal limits including TSH, T4, BUN, and creatinine. After 2 weeks, the patient started to develop acute mental status changes, becoming more confused and lethargic and a repeat sodium level was taken and was 125 MEQ/L. A medicine consult was called and assessed the likely cause of the hyponatremia as being due to medication since the patient did not have any significant GI symptoms or polydipsia. A provisional diagnosis of bupropion induced hyponatremia was made and the medication was stopped. The patient was subsequently started on mirtazapine 7.5 mg Qhs. Five days after stopping the bupropion, the sodium level increased to 130 MEQ/L and then to baseline at 135 MEQ/L in about ten days after discontinuation of bupropion (Figure 1). The patient responded well to mirtazapine with reduction in depressive symptoms, increased appetite, and weight gain.

## 4. Discussion

Antidepressants, including the selective serotonin reuptake inhibitor (SSRI), serotonin norepinephrine reuptake inhibitor (SNRI), and tricyclic antidepressants (TCA), can cause hyponatremia [1, 2]. It is hypothesized that serotonin induces an increase in ADH which is mediated by the hypothalamic serotonin receptors and that the greater the potency of the drug to inhibit the reuptake of serotonin, the greater the chance of hyponatremia [3].

A study found that the serotonergic antidepressants (SSRIs, venlafaxine, and clomipramine combined) were at a greater risk for causing hyponatremia when compared with other antidepressants (OR 3.96 versus 1.78, resp.) [4].

Bupropion's primary pharmacological action is to inhibit norepinephrine-dopamine reuptake. Although bupropion does not act on serotonergic receptors, there are only a few case reports describing how bupropion can be associated with hyponatremia [5–7]. Animal studies have shown that both serotonin and noradrenaline can increase ADH secretion by stimulating serotonergic and *α*-adrenergic receptors [8, 9]. Bupropion therefore may cause hyponatremia by the noradrenergic stimulation of vasopressin release.

Estimates of hyponatremia associated with SSRI and SNRI use vary from 0.5 to 32% [7]. Aging is one of the most significant risk factors for SSRI induced hyponatremia and the incidence rates range from 12% to 33% in the elderly population [10–12]. The cause of this may be due to altered renal function, increased ADH secretion, increased sensitivity to ADH, presence of concomitant illnesses, and other medications that contribute to hyponatremia [10]. Other risk factors include diuretic drug use [13], recent or current pneumonia, lower base line serum sodium levels, and lower body mass index [11].

The German drug-surveillance program in psychiatric inpatients could not detect a single case of hyponatremia due to mirtazapine alone [3]. Also, as per Jung et al., there were no cases of hyponatremia with mirtazapine as compared with 8% on SSRIs and 4% on venlafaxine [14]. It has also been described that mirtazapine has been successfully used in treating patients in whom hyponatremia had developed previously while on an SSRI [15, 16].

Clinicians should be aware of the higher risk and signs of hyponatremia associated with certain antidepressants, as clinical symptoms of hyponatremia can be misinterpreted as a worsening of the primary psychiatric illness.

It is recommended that sodium levels should be checked in all elderly patients exhibiting abrupt or unexplained changes in mental status (e.g., lethargy or confusion) at any time during treatment with an SSRI, SNRI, and NDRI medication [12]. Some recommend a routine serum sodium level at start of therapy and within the first 2 weeks after initiating such treatment, especially if the patients are older, are female on diuretics, have low BMI, and have a baseline plasma sodium level lower than 138 MEQ/L [3, 12, 17, 18].

In our patient, the hyponatremia resolved once the bupropion was stopped and subsequently had a good clinical response to mirtazapine with no further hyponatremia throughout the course of the hospital.
